# Stroke rehabilitation in urban and rural settings in the Philippines: Protocol for an interview and visual elicitation study

**DOI:** 10.1371/journal.pone.0307330

**Published:** 2024-08-22

**Authors:** Sarah Ann Buckingham, Alyssa Marie Dar Juan, Sara Demain, June Ann De Vera, Myrna Estrada, Lorraine Hermosura-Faeldon, Bridie Kent, Maria Teresa Sharon Linog, Roy Francis Navea, Fiona Jones, on behalf of the TULAY project team

**Affiliations:** 1 School of Nursing and Midwifery, Faculty of Health, University of Plymouth, Plymouth, Devon, United Kingdom; 2 Institute of Biomedical Engineering and Health Technologies, De La Salle University, Manila, Philippines; 3 School of Health Sciences, University of Southampton, Highfield, Southampton, United Kingdom; 4 Department of Physical and Rehabilitation Medicine, De La Salle Medical and Health Sciences Institute College of Medicine and University Medical Center, De La Salle University, Dasmarinas, Cavite, Philippines; 5 Physical Medicine and Rehabilitation Department, Las Piñas General Hospital and Satellite Trauma Center, Manila, Philippines; 6 Research Institute for Mindanao Culture, Xavier University Ateneo de Cagayan, Cagayan de Oro City, Philippines; 7 Population Health Research Institute, St Georges University of London, London, United Kingdom; 8 Bridges Self-Management Limited, London, United Kingdom; PLOS: Public Library of Science, UNITED KINGDOM OF GREAT BRITAIN AND NORTHERN IRELAND

## Abstract

**Introduction:**

There is a lack of community-based rehabilitation for stroke in the Philippines, and research on this topic is limited. Different challenges may be encountered in urban and rural settings. The aim of the Tulong, Ugnayan ng Lingap At gabaY (TULAY) project is to develop a context-appropriate, community-based stroke support programme, consisting of self-management and training resources to augment the rehabilitation and recovery process. An important stage in the development of this programme is to qualitatively explore the experiences and needs of all stakeholders.

**Materials and methods:**

Using co-designed and evidence-based topic guides, in-depth semi-structured interviews will be conducted with people living with stroke, household carers and care providers. We will aim to gain a representation of different regions (within Luzon, Visayas, and Northern Mindanao), socioeconomic levels, and urban and rural locations. For people with stroke and household carers, interviews will be supplemented by auto-photography and visual elicitation to widen access for those that prefer to share their experiences visually or have communication problems. An interpretivist paradigm will be applied across all interview data and the consolidated criteria for reporting qualitative research (COREQ) will be followed. Thematic analysis will be undertaken using guidance by Braun and Clarke.

**Discussion:**

To our knowledge, this study is the first of its kind in the Philippines. It has several methodological strengths, including the capture of perspectives from multiple stakeholders in diverse settings, the inclusion of people with communication difficulties, use of visual methods, and analysis in the native language. The findings will have various applications, including the potential to influence policy, practice and guidelines, and to inform the development of the TULAY stroke support programme.

## Introduction

Low-middle income countries have a high burden of stroke, with rates of disability and death greater than those in higher-income countries [1–3]. In the Philippines, stroke is a primary cause of morbidity and the second leading cause of mortality, equating to approximately 87,402 deaths per year [4]. The majority of the cost of healthcare is borne out-of-pocket by patients and their families, resulting in limited availability and accessibility of services for lower- and middle-income groups [5, 6]. There is a lack of established community-based care facilities in the Philippines, and a scarcity of qualified staff to deliver rehabilitation [5]. Audit and review findings suggest geographical, sociodemographic and economic inequities in healthcare provision and access, with greater challenges in rural, isolated and deprived areas [5–8], but a more thorough understanding of the lived experiences and challenges faced by people with stroke and their families and communities is needed.

There is an urgent need to explore and understand experiences across different regions of the Philippines in order to understand the support, training and interventions that are needed. This can only be achieved through an understanding of the perspectives of stakeholders and the specific contextual factors that affect the Philippines. The aim of the TULAY project (Tulong, Ugnayan ng Lingap At gabaY) is to develop a community-based stroke support programme, consisting of self-management and training resources, to enhance the rehabilitation and recovery process.

The aim of this study is to explore the lived experiences and unmet needs of people living with stroke, their household carers, and professional care providers, deepening our understanding of the nature and quality of existing stroke rehabilitation and care services in urban and rural settings. This will provide qualitative data to complement and build on the knowledge of stroke services attained in our national survey (data collection completed May 2024, analysis ongoing). The findings and data collected will be used to inform the co-design of the novel stroke support programme to ensure that it is culturally relevant and tailored to the needs of stakeholders.

## Materials and methods

### Study design

A descriptive qualitative design will be used in this study. Traditional semi-structured interviews will be used. These will be complemented by auto-photography and visual elicitation to enhance the quality and richness of data.

An interpretivist paradigm will be applied to gain an understanding of individual experience from the perspective of the person. This approach recognises the importance of subjective interpretation, perceptions and meaning attached to experiences [9].

The consolidated criteria for reporting qualitative research (COREQ) [10] were used in the development of the protocol and will be followed throughout the study (S1 File).

### Participants

There will be three categories of participants, with broad inclusion criteria:

1. *People living with stroke*: Any adult individual aged 20 years and above (as defined by the World Health Organization [11]) with stroke. People with more than one stroke and other co-morbidities may participate. People with aphasia (who are often excluded from stroke research) will be included where possible, but people lacking capacity will be excluded.
2. *Household carers*: Relatives, neighbours or friends of people living with stroke who define themselves as providing care or support. Carers must be aged 18 years and above. Carers bereaved within the last five years may be included.
3. *Care providers*: All care providers who have worked with at least one person with stroke in the past 12 months will be eligible to participate. Hospital-based professionals (e.g. clinicians) and community-based professionals (e.g. Municipal Health Officers (MHOs); Barangay Health Workers (BHWs); Nurse Deployment Program (NDP) nurses; midwives; social welfare officers; support workers) may be included. Non-certified health providers (e.g. faith healers) may also be interviewed to gain insight into local and traditional practices.

### Recruitment and sampling

Participants will be purposively selected from a database containing details of those who completed the TULAY national survey and expressed a willingness to participate in subsequent interviews. These participants were initially recruited using cluster random sampling. If necessary, this sample may be supplemented by snowballing and ‘word of mouth’ using contacts of the project team; this may include community groups, the TULAY Steering Committee, and Patient and Public Involvement and Engagement (PPIE) group.

Previous health service utilisation research in the Philippines highlighted inequities according to socioeconomic status, religion, and geographical location or distance from facilities [6–8]. Therefore, three key purposive criteria will be used to select people living with stroke and household carers from the database of survey respondents:

1. Geographical area and region. A number of different regions and municipalities will be included within Luzon (National Capital Region and CALABARZON or Region IV-A), Visayas (Western Visayas—Region VI and Central Visayas—Region VII) and Northern Mindanao (Region X). Islands and geographically isolated and disadvantaged areas (GIDAs) will be included.
2. Socioeconomic level (high vs. low). This will be determined by individual indicators as reported by survey respondents (e.g. education and income) and economic indicators (i.e. municipality annual income generation levels 1 to 6, as defined by the Philippine Statistics Authority [12, 13])
3. Urban vs. rural areas. As reported by survey respondents (or where not reported, as defined by the Philippine Statistics Authority [14]).

People with different health statuses and severities of stroke will be included. Data collection will continue until thematic saturation is reached (i.e. no new themes are being identified in interviews), so it is not possible to determine the precise number of interviews. However, it is anticipated that there will be approximately 20 to 30 interviews with people with stroke and a similar number of interviews with household carers.

The same sample selection scheme will be used for care providers. This will enable inclusion of a wide group of health services from different regions across both urban and rural settings, with an estimated sample size of 20–30 participants.

### Informed consent

**Fig 1:**
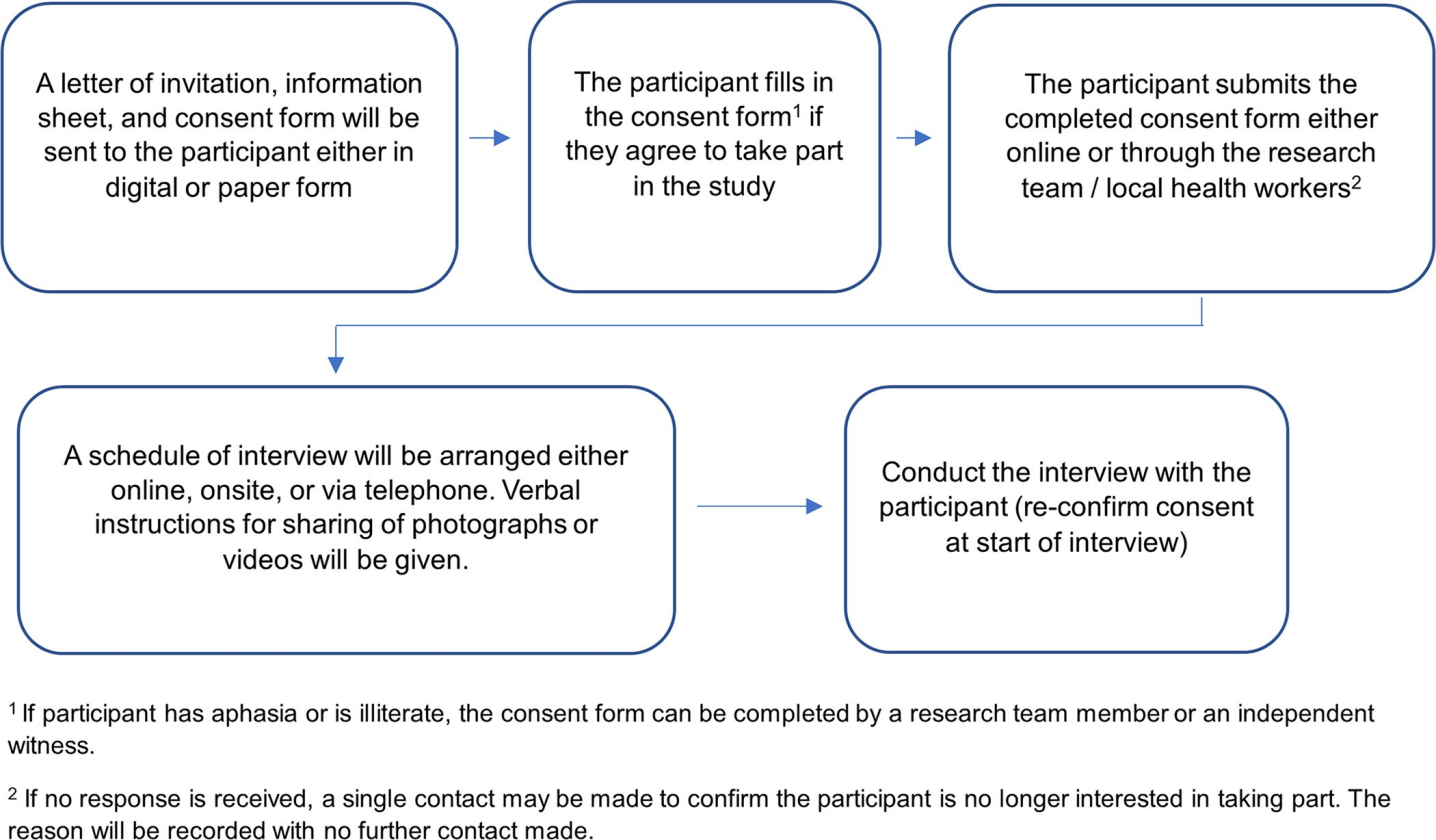
Consent process for interviews.

The process of obtaining consent is shown in Fig 1.

Selected participants will be provided with a letter of invitation and information sheet that will explain the purpose of the study, together with a consent form. The consent form may be completed either in paper or digital form; online consent forms will be completed using Jisc version 3 (onlinesurveys.ac.uk). Documents will be distributed and collected by members of the research team or through the assistance of the NDP nurses and midwives, aided by the local MHO. If the participant agrees to participate, they will fill in the consent form. When consent is received, a member of the research team will contact the participant to arrange the interview at a convenient time and will give instructions regarding sharing of photographs or videos. Consent will be re-confirmed at the beginning of the interview.

Consent forms will contain separate items to indicate consent to participate in an interview, for audio-recording of the interview, for video-recording of the interview, and to allow photographs or videos provided to be used in the production of the TULAY stroke support programme (i.e. images to depict stories and experiences of stroke that can be shared and help other stroke survivors and families). The form will also capture participants’ interest in taking part in future co-design workshops for the TULAY programme.

Although the process in Fig 1 is expected to be followed for most participants, due to the geographically dispersed nature of the study and characteristics of the study population, a flexible approach to gaining consent will be taken. For example, where it is difficult to access the participant’s location, the consent form may be completed on the same day as the interview. In these instances, the researcher will ensure sufficient time is given to understand the study information and decide whether to participate. In circumstances where it is not possible to complete a paper or online consent form (such as participants in isolated regions without internet access), audio-recorded verbal consent may be obtained. For participants with aphasia, capacity will be assessed by a member of the research team, who will establish ability to understand and retain study information and follow a two-step command (e.g. close eyes and nod head) to indicate consent. For these participants, and for those who are illiterate, the consent form will be completed in the presence of the researcher and/or an independent witness.

### Interviews

Individual interviews with people living with stroke, their household carers, and care providers will be conducted online (e.g. via Zoom), via telephone or onsite. The method and location will be selected based on practicality, accessibility, privacy, safety and security, and preferences of the interviewee. Interviews may take place in participants’ homes, workplaces, or other public places such as barangay (village) halls. Interviews will be conducted in the language preferred by the interviewee; this is likely to be English, Tagalog, Cebuano or other regional dialects.

Interviews will be carried out by qualified researchers and research assistants (RAs) in the Philippines who are trained and experienced in qualitative research and working with people with chronic conditions. Interviewers will be male and female and of a range of ages, and some have prior or current health professional roles including physiotherapy and public health practice. All interviewers are working on the TULAY project and have a professional and/or personal interest in stroke rehabilitation and care. Interviewees will be unknown to the interviewers (and vice versa) prior to study commencement. Before the interviews, briefings will be conducted to ensure familiarity with the protocol and ethical considerations.

Where possible, people with stroke and their carers will be interviewed separately; this will encourage people to speak openly and candidly and avoid unnecessary distress when listening to the carer or cared for person’s concerns [15]. Interviews with the person with stroke and their carer will be conducted by the same interviewer where possible. For people with aphasia, to support communication, they may be interviewed in the presence of a family member or carer, and visual methods such as photographs, timelines and hand signals will be used.

The interviews will be semi-structured based on topic guides (S2–S4 Files). Topic guides were informed by earlier stages of the TULAY project, including a scoping literature review and national survey. The content has been co-created with the TULAY PPIE group and reviewed by the wider project team. Topic guides will be piloted and as interviews progress, they may be further iteratively revised based on findings.

The topic guides for each group of participants (people living with stroke, household carers and care providers) have been informed by our PPIE group and include the following broad topics:

- Experiences of stroke, rehabilitation and care
- Barriers and enablers to stroke care and rehabilitation including self-management (or supporting self-management)
- Recommendations for improved stroke services, the TULAY programme, and tips for other people with stroke/household carers/care providers

The interviewer will cover as many topics as possible, but the order will be flexible and open-ended questions will be asked. This will enable a deep understanding of the participant’s experiences and subjective viewpoint, in line with the interpretivist approach. The interviewer may use prompts and probing such as, “Tell me more about…” throughout the interview. Reflecting and clarification (e.g. “So you said… have I got that right?”) and non-verbal communication (e.g. nodding, use of silence) will be used to enhance the interview. Interviewers will maintain a neutral stance and will avoid disclosing their own views and experiences during the interview. At the end of the interview, the interviewer will verbally summarise the content of the discussion to check their understanding and invite the participant to add anything they would like to share.

Interviews are expected to last between 30 minutes and one hour. Interviewers will be mindful of the physical and psychological status of the interviewee, and care will be taken to ensure that sufficient breaks are included if the need arises.

Following each interview, the interviewer will collate detailed field notes. These will include observations of context and setting; details of how the interview was conducted; reflection on their own performance and potential influence on the interview; how the interviewee responded to the questions; and initial summary and thoughts on points arising from the interview.

### Auto-photography and visual elicitation

Auto-photography and visual elicitation will be used in interviews with people with stroke and household carers. These participants will be invited to optionally provide photographs or videos that they have taken to illustrate their experiences, journey to recovery, and participation in life after stroke. The supplied media may be digital or non-digital and may be shared prior to or during the interview. Auto-photography captures the world through the participant’s eyes and visual (photo or video) elicitation involves using the images to generate discussion in the interviews [16]. Where participants have opted to provide a photograph or video, this may be a starting point for discussion following introductions. As an alternative to providing a photograph or video, participants may be given the option to draw or show something that represents their journey to recovery, life after stroke, or experience of caring (which will then be photographed by the interviewer).

Visual methods have several advantages; they can help to build rapport and create a common understanding between the interviewer and the interviewee, particularly where there may be cultural or language differences as in the TULAY project. This provides greater accessibility and ways of communicating for participants with aphasia who are less able to express themselves verbally. Visual methods allow participants to talk about what matters to them, while enhancing the depth and richness of data and increasing validity and rigour [16, 17]. A further benefit is that with participants’ consent, the media may be used to illustrate experiences and self-management strategies for the new TULAY programme; this method has been used extensively in the UK when co-designing and refining self-management resources and training [18].

### Data management and analysis

All interview participants will be assigned a unique alphanumeric identifier (participant ID) to preserve anonymity, with interview files and media named accordingly with date and file type. Files will be stored in a restricted access SharePoint folder linked to a separate password-protected database of participant contact details. Data will be stored and managed in accordance with University policies and data protection laws, including the Philippines Data Privacy Act 2012 and Implementing Rules and Regulations [19], and the General Data Protection Regulation in the UK [20].

Interviews will be transcribed verbatim in the language they were conducted in. Following transcription, all identifiable information (e.g. individual names, place names) will be removed. Pseudonyms will be used in analysis and reporting of findings.

Data will be analysed using thematic analysis, which is compatible with the interpretivist paradigm. The guidance of Braun and Clarke [21, 22] will be closely followed and NVivo 14 software [23] will be used to organise and code transcripts. Both inductive and deductive analysis will be used, enabling the inclusion of themes arising from the data while drawing on categories and concepts within the topic guides. Analysis will be carried out collaboratively by researchers in the Philippines and UK, assisted by regular meetings and online tools such as Jamboard (jamboard.google.com) or Miro (miro.com) to facilitate sharing of ideas.

Following familiarisation with transcripts, a sample of two interviews in each group (people with stroke, household carers and care providers) will be coded separately by UK and Philippines researchers and then discussed. This will enable inter-coder reliability to be checked and will allow the initial coding structure and preliminary themes to be agreed on. To facilitate this process, any Filipino dialect transcripts in this sample will first be translated into English. The translation will be checked by a second person to ensure accuracy. Care will be taken to preserve meaning, for example by retaining phrases unique to the Filipino language and adding English definitions and explanations of terms.

The remainder of the interviews will be analysed in the native language by two or three researchers in the Philippines in consultation with the UK research team members. Language attributes and language-specific features within NVivo will be utilised to aid analysis in different dialects. Any discrepancies in interpretation will be discussed with additional team members with qualitative expertise.

Photographs and videos provided by participants will be imported into NVivo; although the main purpose of these is to stimulate discussion in interviews, they will be coded with short descriptive and analytic notes to aid organisation and clarity. This process will be guided by methods used in photographic analysis [17, 24, 25]. Field notes will also be used to support the analysis process; they may add to richness or completeness of data and facilitate the development of analytic (higher level) themes.

A thematic concept map will be developed for each group of interviews (people with stroke, household carers and care providers); this will include identified themes, their descriptions and codes. When this is complete, we will explore any cross-cutting themes and similarities and differences in perspectives and identified needs and priorities between the different groups. We will also explore differences in the quality of services for people with stroke in rural and urban areas.

Reported findings may include verbatim quotes, translated quotes, and visual materials (photographs and videos). Quotes that allow identification of the participant will be avoided. Visual materials that do not disclose the identity of the participant will be given preference in reporting; where this is not possible, the degree of anonymity will be agreed between the researcher and participant and documented.

Several measures will be taken to ensure quality in data collection and analysis. Rigor and trustworthiness will be achieved by using interview topic guides with clear definitions of terms used, and by using a combination of visual and written data sources. A clear description of methods used and an audit trail to source data will provide dependability. Credibility will be provided through triangulation of themes within the wider project, including feedback from stakeholder and PPIE representatives. To ensure transparency and to add to depth and richness of data, all researchers will document reflections and observations made whilst coding, and meet regularly to share thoughts, ideas and reflections.

### Ethics and safety

Ethical approval for the TULAY project has been granted by the Philippines Department of Health Single Joint Research Ethics Board (SJREB) (Ref: SJREB-2023-85) and the University of Plymouth Faculty of Health Research Ethics and Integrity Committee (Ref: 2024-4703-5965). The standard ethical principles for studies involving human participation, including the Declaration of Helskinki, will be observed in this study.

Voluntary participation, informed consent, and anonymity and confidentiality are described earlier in this paper. Participants will be clearly informed of their right to withdraw from the study at any time without any consequences. They may request removal of their interview data up to the point of analysis and may request removal of any data provided for use in the TULAY support programme at any time prior to completion of the resources.

To ensure inclusivity and understanding, all written information will be provided in the main local dialect of the Region. Paper versions of all participant documents will minimise the risk of digital exclusion. Simplified versions of the information sheets will be available for people with stroke and aphasia.

Participants who have completed an interview will be given tokens of appreciation, in kind, amounting to at most 500 Philippine pesos. Risks of participation are considered negligible. However, if a participant becomes distressed or uncomfortable during the interview, the interview will be paused or terminated. If there are concerns related to participants’ safety and wellbeing, we will inform the local MHO. Participants may be signposted to relevant support agencies (with details included in the information sheets).

The safety of the research team is also paramount. For onsite interviews, a risk assessment will be carried out. A lone working policy will be in place for researchers who are interviewing participants alone. Researchers will be trained in mental health psychosocial support for themselves as well as the participants. In regions of conflict and in the event of natural disasters or extreme weather events, national and local guidance and university protocols will be followed.

### Patient and public involvement and engagement

The TULAY project has a PPIE group that includes people living with stroke and other physical disabilities. The group has reviewed the study protocol and provided feedback on the structure and content of the interview topic guides, in addition to all participant-facing documents. The group will be consulted throughout the study and their views will be sought if any unexpected issues arise or if minor changes to the protocol are necessary.

### Study status and timelines

Recruitment and data collection for this study are expected to begin in June 2024. Data analysis will be on a progressive mode as soon as data are available, with completion of the study in March 2025 and findings published after this date. The TULAY project is expected to finish by September 2026.

## Discussion

### Contributions

To our knowledge, this is the first qualitative study to explore stroke rehabilitation and care in urban and rural settings in the Philippines, and to capture multiple perspectives of people living with stroke, household carers and care providers. The study has several methodological strengths. The use of visual methods will enhance depth, richness and quality of data. The broad inclusion criteria will ensure that diverse geographical, socioeconomic, and urban and rural settings are represented, and the experiences and views of people with aphasia. Further, the interviews will be analysed in the native language by researchers in the Philippines to ensure meaning and context are preserved, but with support from experienced qualitative researchers in the UK who are able to offer an independent external perspective.

This protocol may serve as a model and guide for researchers and practitioners undertaking research in a similar context, for example studies involving international collaboration and remote working, qualitative research conducted in different languages, and research in low-middle income countries.

### Limitations

As a qualitative study, the findings will be context-specific and may therefore not be generalisable to other populations or settings outside of the Philippines. Although measures are in place to overcome geographical and digital barriers, it is likely that there will still be some difficult-to-reach individuals who it will not be possible to interview, such as those who have not engaged with healthcare services. Despite these limitations, it is envisioned that the findings will inform policy, practice and future planned research that will lead to better community rehabilitation and care services in the Philippines.

### Dissemination

Four main dissemination routes are planned for the outputs of this study:

- The study report will inform the Local Government Units (LGUs) and the joint Philippines’ DoH and Philippine Academy of Rehabilitation Medicine (PARM) discussion group meetings, providing recommendations for further actions and informing Clinical Practice Guidelines on community-based rehabilitation.
- Lay summaries will be translated in different dialects and shared with local support groups for people with stroke and other disabilities.
- Publication(s) of findings will be submitted to a peer reviewed journal.
- A concept map, summaries, recommendations, and data and media gathered will inform development of the TULAY support programme (self-management and training resources).

## Supporting information

S1 File Research checklist (COREQ). (PDF)

S2 File Interview topic guide: People with stroke (English version). (PDF)

S3 File Interview topic guide: Household carers (English version). (PDF)

S4 File Interview topic guide: Care providers (English version). (PDF)
