# Supplementary material for: Stroke rehabilitation in urban and rural settings in the Philippines: Protocol for an interview and visual elicitation study
Source: PLoS One. 2024 Aug 22;19(8):e0307330. doi: 10.1371/journal.pone.0307330 (PMC11340961; doi:10.1371/journal.pone.0307330)
Supplement: S2 File — (PDF) [file pone.0307330.s002.pdf]

Interview topic guide: People with stroke (English version)

Interviewer introduction:

- Introduce self and TULAY project
- Briefly explain the purpose of the interview and what topics will be covered
- Check how much time the participant has available
- Check they have read and understood the study information – do they have any questions?
- State the interview is voluntary and they can withdraw at any time or request removal of data before it is analysed
- Highlight data protection – data (contact details, interview recording, photos/videos) will be stored securely; personal details will not be shared outside the research team; any names/local places/hospitals you mention will be removed from interview transcripts and reports
- Check consent form has been completed – if not, do this now
- Re-confirm consent for video- or audio-recording the interview (log interview start and end time) and start recording

Notes for interviewers and notetakers: Focus on the questions (middle column) and use the prompts as necessary. Give the participant time to think of their own answer before giving examples. **If the participant can do the interview without the presence of the carer, this will be preferred and encouraged.** *If there are limitations and the carer must be present during the interview,* consider the wording and delivery of the questions and take into account body language and hesitancy to answer.

Topic	Questions	Prompts
**BACKGROUND AND EXPERIENCES***		
About you and the impact of your stroke	Could you tell me a bit about yourself and how long it has been since you experienced a stroke?<br><br>Can you say a bit about where you live and who you live with?<br><br>In what ways has the stroke affected you and the things you enjoy doing? (for example, your ability to move around or work)?	E.g. Occupation and life before stroke; time since stroke; other health conditions<br><br>Fully or partially recovered?<br><br>Urban or rural; type of house (e.g. two-storey); live alone or with family/friends; presence of carer<br><br>(Guide): *(Broken down into thematic functions)* How has your stroke impacted you..<br>Physically?<br>Financially? (e.g. had to give up work, source of income before and after stroke, cost of treatment)<br>Socially/psychologically?<br>Cognitively?<br>**Probe
**Photo/video elicitation *(refer back to throughout the interview)***	<u>**Note:**</u> Participants are invited to prepare photos and videos that reflect what is important and significant to them right now or that signifies their journey to recovery and life after stroke<br><br>Option A: *If Participant has photo(s) or video(s) to share*<br><br>Can you describe/explain the photo or video you have provided?<br><br>Option B: *If Participant has no photos or videos prepared, interviewer may ask participant to*	 <br><br><br>Option A: What does it mean? Why did you choose this image/video?<br>Why is this important to you? (ask further probing questions as appropriate)<br><br>Option B: How do you feel about this drawing/item? (ask further

	draw or show something that the interviewer can take a picture of, i.e. something that represents their journey to recovery and life after stroke. "If you were to take a photo right now of what is meaningful to you, what would it be?"<br>**Take a photo of this with the participant's consent.**	probing questions as appropriate)
Experiences of stroke care and rehabilitation	Could you talk through the treatment and care you received from when you first had a stroke to when you went home and to the present day?<br><br>Did you take part in or receive any rehabilitation programmes? If so, can you describe these?<br><br>*If rehab was received:*<br>In your opinion, how effective were these rehabilitation programmes in helping you recover?<br><br>Did you receive any other care (e.g. traditional care, faith healers)?	What treatment?<br>When? Duration?<br>Who provided this?<br>In what settings (e.g. inpatient, outpatient, community)?<br><br>*Ask about care:*<br>*Immediately post-stroke*<br>*After discharge/home care*<br>*Present care*<br><br>E.g. therapies; setting; individual or group; types of activities (social/cognitive/physical); duration; cost<br><br>What worked/did not work?<br>> Health outcomes (e.g. mobility, daily tasks, speech); speed of recovery *(probe and expound as needed)*<br><br>How did these types of care help you? Were there any positive or negative effects on you after receiving this type of care?
**BARRIERS AND ENABLERS**		
Barriers and enablers to accessing and receiving	What challenges did you face in accessing or receiving stroke rehabilitation or care?	Prompt: *(Broken down into thematic functions)*

rehabilitation and care	Are there physical challenges you face/d while receiving stroke rehabilitation or care?<br><br>Are there accessibility challenges you face/d while receiving stroke rehabilitation or care?<br><br>Do you think that where you live (i.e. region, urban or rural area) had any effect on the treatment you received and your recovery?<br><br>What helped you to access care and rehabilitation (or what would make it easier to access in the future)?<br><br>Are there financial challenges you face/d while receiving stroke rehabilitation or care?<br><br>Are there social and psychological challenges you face/d while receiving stroke rehabilitation or care?<br><br>How has your mindset and experiences contributed to your coping and recovery?<br><br>Overall, how do you feel that these challenges have affected your recovery?	> Probe: Health conditions; mobility constraints<br><br>> Probe: Lack of facilities and rehab centers; geographical challenges; lack of transportation<br><br>Better or worse and why? E.g. distance from facilities; travel time and expense; terrain; availability of transportation<br><br>E.g. support from family/health workers/charities/community; public transport; PhilHealth insurance; online support.<br><br>>Probe as necessary<br><br>> Probe on stigma, fear of labelling? mental health issues (feelings of depression, anxiety, probe on fears…)<br><br>E.g. withdrawn from social life; lack of collectedness; help-seeking behaviours
Social support	Who has supported you in managing life after your stroke?	Support from family and friends; health and social care professionals; faith healers; charities; local community or village groups; stroke support groups (in person or online)

	What types of support did they provide?<br><br>*If you attended a support group,* were you introduced to it or did you seek it out?<br><br>Do you feel that you want to talk to someone other than those in your household for emotional support?	Physical; practical; psychological/emotional; financial; spiritual<br><br>E.g. Psychologist, professional, faith healer, spiritual adviser...
Knowledge, skills and confidence in self-management	Where did you (or do you) get information about stroke and recovery? Was there anything you wanted to know that would have helped you? (knowledge)<br><br>What skills do you think are important in managing life after a stroke? Are there skills you think are important to develop? (skills)<br><br>What (if anything) would help you feel more confident in managing your life after a stroke? (confidence)	E.g. health workers; support groups; online; other sources of information<br><br>E.g. coping with stress; problem-solving; goal-setting; decision-making; self-management<br><br>E.g. more information; support from health workers; sharing experiences with others
Other barriers and enablers to participation in life after stroke	Are there any other factors that have made your recovery and participation in life after stroke more difficult?<br><br>Is there anything we have not discussed that has helped you to recover and participate in life after a stroke?	
	**RECOMMENDATIONS**	
General improvements to services supporting life after stroke	Based on your experience, what improvements can be made to the stroke rehabilitation and care systems in your area?	E.g. access to healthcare facilities; more support groups; training of health workers; public awareness; improved funding

Recommendations for TULAY programme	We are looking to design a new self-management programme for people with stroke in the Philippines.<br><br>What would you like it to include?<br><br>What resources or tools would help you?<br><br>Would you prefer:<br>Written or online resources?<br>Website or app?<br>Guided by a care professional or work through yourself/with a family member?<br>Group or one-to-one therapy?	E.g. types of activities (exercise, music, relaxation); shared experiences; meeting other people with stroke; progress tracker or diary; stories; videos; pictures; livelihood opportunities; maps of facilities; carers; social welfare help/information.<br><br>Probe on preferred content and format. Any other suggestions?
Top tips and advice for other people with stroke	What are your top tips for managing life after stroke to your fellow stroke survivors?<br><br>What would you say to someone who has recently experienced a stroke knowing what you know now?	
Additional comments	Do you have any final thoughts or additional comments?<br><br>Do you have any questions for the interviewers?	

**Note for interviewers:* we will have survey data on impact of stroke and treatment/ rehabilitation so try not to spend too long on these questions!

Interviewer summary:

- Summarise key points discussed during the interview.
- Ask if there is anything they would like to discuss further and state that you are available to discuss the study, especially if there are any issues that arose or discussion points that may have caused distress.

- Check they are still happy for the recorded interview/photos/videos to be used for the purposes stated on the consent form.
- Thank the participant for their time and insights. Give token.
- Ask if they would like to receive a summary of study findings.
