# Supplementary material for: Stroke rehabilitation in urban and rural settings in the Philippines: Protocol for an interview and visual elicitation study
Source: PLoS One. 2024 Aug 22;19(8):e0307330. doi: 10.1371/journal.pone.0307330 (PMC11340961; doi:10.1371/journal.pone.0307330)
Supplement: S3 File — (PDF) [file pone.0307330.s003.pdf]

V1.2 01/05/2024

Interview topic guide: Household carers (English version)

Interviewer introduction:

- Introduce self and TULAY project
- Briefly explain the purpose of the interview and what topics will be covered
- Check how much time the participant has available
- Check they have read and understood the study information – do they have any questions?
- State the interview is voluntary and they can withdraw at any time or request removal of data before it is analysed
- Highlight data protection – data (contact details, interview recording, photos/videos) will be stored securely; personal details will not be shared outside the research team; any names/local places/hospitals you mention will be removed from interview transcripts and reports
- Check consent form has been completed – if not, do this now
- Re-confirm consent for video- or audio-recording the interview (log interview start and end time) and start recording

Notes for interviewers and notetakers: Focus on the questions (middle column) and use the prompts as necessary. Give the participant time to think of their own answer before giving examples. For the household carer interview, **the carer and interviewer must not be in the presence of the person with stroke** as this may affect the answers of the carer.

Topic	Questions	Prompts
	**EXPERIENCES**	
About you	Could you tell me a bit about yourself and the person you care for or support?<br><br>What was your occupation before you became the carer of _____?	E.g. Occupation / education; relationship with cared for person; duration of caregiving; current health status of person with stroke<br><br>Probe on their life before they became a stroke carer
**Photo/video elicitation *(refer back to throughout the interview)***	**Note:** Participants are invited to prepare photos and videos that reflect what is important and significant to them right	

	now or their experiences of caring<br><br>Option A: *If Participant has photo(s) or video(s) to share* Can you describe/explain the photo or video you have provided?<br><br>Option B: *If Participant has no photos or videos prepared, interviewer may ask participant to draw or show something that represents their experience of caring for the person with stroke.* **Take a photo of this with the participant's consent.**	Option A: What does it mean? Why did you choose this image/video?<br>Why is this important to you? (ask further probing questions as appropriate)<br><br>Option B: How do you feel about this drawing/item? (ask further probing questions as appropriate)
Experiences of caring for the person with stroke	In what ways do you support __________ (person with stroke)?<br><br>What is one thing you have loved about supporting __________ (person with stroke)?<br><br>What has been the most challenging aspect of supporting __________ (person with stroke)?<br><br>How has supporting __________ (person with stroke) affected your life or wellbeing?<br><br>How has supporting __________ affected the household in general?	Physical; practical; psychological/emotional; financial; spiritual<br><br>E.g. spending more time together; seeing them start to get well<br><br>Anything now or in the past. E.g. change of relationship; resistance from person with stroke; lack of external support<br><br>E.g. work; social life; physical health; finances; emotional wellbeing<br><br>E.g. household members that stopped schooling; forced to take on extra work; ways to augment income

BARRIERS AND ENABLERS		
Overall support	Have you received any formal or informal support or as a carer?<br><br>Have you received any financial support or compensation?<br>***If yes:*** government or non-government?<br>How much compensation?<br>Do you feel that this is sufficient?<br><br>Have you received any social/psychological support as a carer?<br>***If yes:*** who provided this?<br>How could it be improved?<br><br>Do you feel that you want to talk to someone other than those in your household for emotional support?<br><br>Have you received any training in looking after someone with a stroke?<br>***If yes:*** who provided this?<br>How could this support be improved?<br><br>Do you think that where you live (i.e. region, urban or rural area) had any effect on the support you received as a carer?<br>How? Please explain.	Probe on type of support<br><br>E.g. Philhealth; private corp.; civil society organisation groups; charities, etc.<br><br>E.g. Social support groups; wider community groups; other family member support; mental health services; counselling, etc.<br><br>E.g. Psychologist; professional; faith healer; spiritual adviser, etc.<br><br>Probe more on this
Knowledge, skills and confidence as a carer	Where did you (or do you) get information about stroke and caring for someone with	E.g. health professionals; support groups; online; other sources of information

	stroke? Was there anything that you wanted to know that would have helped you? (knowledge)<br><br>What skills do you think are important in your role as a carer? Are there skills you think are important to develop? (skills)<br><br>What (if anything) would help you feel more confident in your role supporting ________ (person with stroke)? (confidence)	 <br><br><br>E.g. communication; listening and understanding; supporting while encouraging independence<br><br><br>E.g. more information; support from health workers; sharing experiences with other carers
Other barriers and enablers	Is there anything we have not discussed that gets in the way of supporting _________ (person with stroke) to do more of what they want to do?<br><br>What would help you to support____________ (person with stroke) in doing more of the things they love?	
**RECOMMENDATIONS**		
General improvements to services supporting people with stroke and carers	Based on your experience, what improvements can be made to the stroke rehabilitation and care systems in your area?	E.g. access to healthcare facilities; more support groups; training of health workers; public awareness; improved funding; greater involvement of family carers.
Recommendations for TULAY programme	We are looking to design a new self-management programme for people with stroke and their families or carers in your community.	

	What would you like it to include?<br><br>What resources or tools would help you?<br><br>Would you prefer:<br>Written or online resources?<br>Website or app?<br>Guided by a care professional or work through with the person with stroke?<br>Group or one-to-one therapy?	E.g. types of activities (exercise, music, relaxation); shared experiences of other carers; meeting other carers; progress tracker or diary; stories; videos; pictures.<br><br>Probe on preferred content and format. Any other suggestions?
Top tips and advice for other carers	What are your top tips for other people like yourself who are supporting a person with stroke?<br><br>What advice would you give to people (like health workers) who are supporting carers?	
Additional comments	Do you have any final thoughts or additional comments?<br><br>Do you have any questions for the interviewer?	

Interviewer summary:

- Summarise key points discussed during the interview.
- Ask if there is anything they would like to discuss further and state that you are available to discuss the study, especially if there are any issues that arose or discussion points that may have caused distress.
- Check they are still happy for the recorded interview/photos/videos to be used for the purposes stated on the consent form.
- Thank the participant for their time and insights. Give token.
- Ask if they would like to receive a summary of study findings.
