# Supplementary material for: Stroke rehabilitation in urban and rural settings in the Philippines: Protocol for an interview and visual elicitation study
Source: PLoS One. 2024 Aug 22;19(8):e0307330. doi: 10.1371/journal.pone.0307330 (PMC11340961; doi:10.1371/journal.pone.0307330)
Supplement: S4 File — (PDF) [file pone.0307330.s004.pdf]

Interview topic guide: Care providers (English version)

Interviewer introduction:

- Introduce self and TULAY Project
- Briefly explain the purpose of the interview and what topics will be covered
- Check how much time the participant has available
- Check they have read and understood the study information – do they have any questions?
- State the interview is voluntary and they can withdraw at any time or request removal of data before it is analysed
- Highlight data protection – data (contact details, interview recording) will be stored securely; personal details will not be shared outside the research team; any names/local places/hospitals you mention will be removed from interview transcripts and reports
- Check consent form has been completed – if not, do this now
- Re-confirm consent for video- or audio-recording the interview (log interview start and end time) and start recording

Note for interviewers: Focus on the questions (middle column) and use the prompts as necessary. Give the participant time to think of their own answer before giving examples.

Topic	Questions	Prompts
**EXPERIENCES**		
About you and your role	Could you tell me about your work role and experience of stroke care or rehabilitation?	Title of role<br>What setting do you work in (inpatient/outpatient/community)?<br>Region<br>Urban vs. rural?<br>What does your work involve?<br>How many people with stroke do you typically see?<br>Do you work alone or as part of a team?<br>How long have you worked with people with stroke?
Overview of stroke rehabilitation and care	Can you give a brief overview of stroke rehabilitation and care services in your areas?	What services are available?<br>Who provides these?

	How typical is this structure compared to other areas?	In what settings (e.g. inpatient, outpatient, community)?<br>Have you worked in another stroke centre before? Describe and compare.
**BARRIERS AND ENABLERS**		
Barriers and enablers to access and receipt of rehabilitation (including regional variation and inequalities)	In your experience, what are the major barriers or challenges faced by stroke survivors in accessing or receiving rehabilitation and care?<br><br>Do you feel that there are any variations or inequalities in how people access and receive care and support after their stroke?<br><br>***If yes:*** Why? What factors contribute to these variations or inequalities?<br><br>How might these challenges or inequalities be overcome?	E.g. lack of knowledge or awareness; lack of facilities (e.g. rehab centres); lack of local support groups; lack of health or social care professionals; lack of family support; lack of PhilHealth coverage/financial capacity<br><br>Who is most likely to access and receive rehabilitation?<br>Geographic differences; urban vs. rural areas; richer vs. poorer areas.<br>Marginalised populations.<br>Age; gender; socioeconomic status; education; health status.<br><br>Availability of healthcare insurance; policy; transport; availability of health and social care professionals.<br><br>E.g. raise awareness; advocacy of primary and secondary prevention; transport; strengthen LGU collaborations with primary health workers; technologies and telerehab; wider PhilHealth availability; online support; self-management programmes.
Knowledge, skills and confidence in supporting self-management	Have you received any training in supporting people with stroke? ***If yes:*** Describe the training<br><br>What guidelines or protocols for stroke rehabilitation and	Yes/no; type of training; where; when; content; duration.

	care do you currently use (if any)? Is there any information that you feel is missing? (knowledge)<br><br>What skills do you think are important in supporting people to participate in life after stroke? (skills)<br><br>How confident do you feel in supporting people with stroke? What (if anything) would help you feel more confident? (confidence)	E.g. national or local guidelines; policies; other sources of information<br><br>E.g. encouraging independence; motivating; listening; coaching.<br><br>E.g. improved guidance; training; interdisciplinary working; collaboration with stakeholders; use of rehabilitation technologies.
Other barriers and enablers to supporting stroke rehabilitation and self-management	Are there any other factors that make it more difficult for you to support people with stroke?<br><br>Is there anything we have not discussed that has helped you to support people with stroke?	E.g. financial; time; resources, location – accessibility; patient compliance; motivations
**RECOMMENDATIONS**		
General improvements to services supporting life after stroke	Based on your experience, what improvements can be made to the stroke rehabilitation and care systems in the Philippines (in the short or long term)?<br><br>Do you know of any examples of innovation or best practice for providing stroke rehabilitation and care within the Philippines?	E.g. access to healthcare facilities; more patient support groups; training of health workers/village workers; standardised guidance; public awareness; improved funding; inter-, multi- and trans-disciplinary working/exchange of best practices; use of telerehab and rehabilitation technologies; expanded PhilHealth coverage for outpatient and community-based patients and services.<br><br>E.g. collaborative programmes; community support groups; online support; telerehab.

	If yes:<br>- Why do you think this works well?<br>- Could it be applied to other areas of the Philippines (or other low-middle income countries)?<br>- Why/why not?	E.g. involvement of families/communities/charities/ stakeholders; varied programmes; choice of activities; creative or innovative ideas.
Training needs for TULAY programme	We are looking to design some training resources for health and social workers who work with people with stroke and their family members in the Philippines in a community-based setting.<br><br>What would you like to see included in these training resources? Are there any particular aspects of supporting people to participate in life after stroke where you would like further training?<br><br>What form of training would you prefer?	 <br><br><br><br><br><br><br>Content of training<br><br><br><br><br><br><br><br><br>E.g. self-guided learning; taught courses; blended approach; online or in-person; group or one-to-one; peer learning; vocational training
Top tips and advice for other care providers	What are your top tips for other care providers who support people to participate in life after stroke?	Providing care within<br>- Health facilities<br>- Community<br>- Household (working with household carers)<br>Focus on rehabilitation / participation in life after stroke.
Additional comments	Do you have any final thoughts or additional comments?	

Interviewer summary:

- Summarise key points discussed during the interview.
- Ask if there is anything they would like to discuss further and state that you are available to discuss the study, especially if there are any issues that arose or discussion points that may have caused distress.
- Check they are still happy for the recorded interview to be used for the purposes stated on the consent form.
- Thank the participant for their time and insights. Give token.
- Ask if they would like to receive a summary of study findings.
